# A Mindfulness-Based App Intervention for Pregnant Women: Qualitative Evaluation of a Prototype Using Multiple Case Studies

**DOI:** 10.2196/58265

**Published:** 2025-01-17

**Authors:** Silvia Rizzi, Maria Chiara Pavesi, Alessia Moser, Francesca Paolazzi, Michele Marchesoni, Stefania Poggianella, Erik Gadotti, Stefano Forti

**Affiliations:** 1 Digital Health Research Centre for Digital Health & Wellbeing Fondazione Bruno Kessler Trento Italy; 2 Istituto Pavoniano Artigianelli Trento Italy; 3 Digital Health Innovation Lab Centre for Digital Health & Wellbeing Fondazione Bruno Kessler Trento Italy; 4 Centre for Digital Health & Wellbeing Fondazione Bruno Kessler Trento Italy

**Keywords:** mindfulness, promoting well-being, pregnancy, eHealth, mHealth, mobile apps, development, usability, user-centered design, well-being, maternal health, digital health, intervention, design, preliminary testing, technology-based, interview, multidisciplinary approach, mother, women, WhatsApp, email, midwife

## Abstract

**Background:**

Pregnancy is a complex period characterized by significant transformations. How a woman adapts to these changes can affect her quality of life and psychological well-being. Recently developed digital solutions have assumed a crucial role in supporting the psychological well-being of pregnant women. However, these tools have mainly been developed for women who already present clinically relevant psychological symptoms or mental disorders.

**Objective:**

This study aimed to develop a mindfulness-based well-being intervention for all pregnant women that can be delivered electronically and guided by an online assistant with wide reach and dissemination. This paper aimed to describe a prototype technology-based mindfulness intervention’s design and development process for pregnant women, including the exploration phase, intervention content development, and iterative software development (including design, development, and formative evaluation of paper and low-fidelity prototypes).

**Methods:**

Design and development processes were iterative and performed in close collaboration with key stakeholders (N=15), domain experts including mindfulness experts (n=2), communication experts (n=2), and psychologists (n=3), and target users including pregnant women (n=2), mothers with young children (n=2), and midwives (n=4). User-centered and service design methods, such as interviews and usability testing, were included to ensure user involvement in each phase. Domain experts evaluated a paper prototype, while target users evaluated a low-fidelity prototype. Intervention content was developed by psychologists and mindfulness experts based on the Mindfulness-Based Childbirth and Parenting program and adjusted to an electronic format through multiple iterations with stakeholders.

**Results:**

An 8-session intervention in a prototype electronic format using text, audio, video, and images was designed. In general, the prototypes were evaluated positively by the users involved. The questionnaires showed that domain experts, for instance, positively evaluated chatbot-related aspects such as empathy and comprehensibility of the terms used and rated the mindfulness traces present as supportive and functional. The target users found the content interesting and clear. However, both parties regarded the listening as not fully active. In addition, the interviews made it possible to pick up useful suggestions in order to refine the intervention. Domain experts suggested incorporating auditory components alongside textual content or substituting text entirely with auditory or audiovisual formats. Debate surrounded the inclusion of background music in mindfulness exercises, with opinions divided on its potential to either distract or aid in engagement. The target users proposed to supplement the app with some face-to-face meetings at crucial moments of the course, such as the beginning and the end.

**Conclusions:**

This study illustrates how user-centered and service designs can be applied to identify and incorporate essential stakeholder aspects in the design and development process. Combined with evidence-based concepts, this process facilitated the development of a mindfulness intervention designed for the end users, in this case, pregnant women.

## Introduction

### Background

In a woman’s life, the transition to motherhood is a time of profound change, requiring reorganizing her identity [1]. A woman’s general well-being is affected by pregnancy, as various changes are expected at the physical, mental, and social levels [2]. Several studies are presented in the literature showing the presence of psychological symptoms in pregnant women. The presence of symptoms of anxiety, stress, and depression are most commonly emphasized [3,4]. Regarding anxiety and psychosocial stress, it is estimated that between 12.2% and 39% of pregnant women may suffer from these conditions [5]. Psychosocial anxiety is defined as an imbalance resulting precisely from the challenges of the new condition [6]. Therefore, a woman’s quality of life and her psychological health are affected by how she reacts to all these changes. The vulnerable period of pregnancy and early motherhood also often involves increased worries and fears related to managing the impending changes associated with childbirth and parenting [7,8]. Woods and colleagues [9] found a spectrum of stress symptoms ranging from 6% (91/1522; high levels) to 78% (1190/1522; medium-low stress levels). Only 16% (241/1522) of the sample reported nothing. Another clinically significant condition in pregnant women is perinatal depression [10]. This occurs in 14.5% during pregnancy and reaches up to 49% in the first year after delivery [3].

Although psychoeducational interventions aimed at the psychological well-being of pregnant women are still scarce, ensuring a positive pregnancy experience is crucial, as it lays the foundation for cognitive motivation and a healthy future relationship between mother and child [11]. The use of digital tools represents a promising solution. Indeed, digital interventions offer accessibility and flexibility and overcome social stigma, making them an optimal choice for women seeking support [12,13].

Recognizing the critical need to promote psychological well-being during the transition to motherhood, especially in the digital age, becomes imperative.

According to Kabat-Zinn [14], mindfulness is defined as “the awareness that arises from paying attention, on purpose, in the present moment, and non-judgmentally.” This practice involves actively noticing one’s thoughts, emotions, and bodily sensations and fostering an attitude of openness and acceptance. Kabat-Zinn [15] emphasizes the significance of mindfulness in reducing stress and enhancing overall well-being. Mindfulness practices enable pregnant women to manage stress, anxiety, and depression actively, equipping them with valuable skills for various situations, including childbirth [14,16-19]. For this reason, mindfulness interventions delivered through web-based platforms, for example, have shown promise in improving the psychological well-being of pregnant women [17,20,21].

Therefore, psychoeducational interventions using mindfulness-based digital solutions have significant potential to promote psychological well-being, foster women’s psychological adjustment during physiological pregnancies, and prevent clinically relevant symptoms [22].

This study illustrates the creation of a tailored mindfulness intervention for pregnant women using technology and a dedicated mobile app. Building on established mindfulness concepts proven effective in face-to-face interventions [23-25], the study adopted a user-centered design approach to ensure alignment of the intervention with users’ needs and contextual use [26,27]. The fundamental principle of user-centered design is to actively involve users in the design and development stages, ensuring that they influence the evolution of the product [28].

The user-centered design approach ensures a holistic understanding of user perspectives by incorporating various methods that facilitate user participation at different development stages and involvement levels [29]. In addition, the study incorporated service design principles, emphasizing whole ecosystem experiences, such as how and when it is used, by whom, and where, rather than focusing exclusively on the end product [30]. This fusion of user-centered and service design approaches aimed to involve stakeholders, including pregnant women, health care providers, mindfulness and communication experts, psychologists, and midwives, throughout the design and development process. The overall goal was to create a final product that was easy to use, practical, engaging, and motivating, and fit seamlessly into the larger context of daily life and the challenges faced during pregnancy.

### Mindfulness-Based Childbirth and Parenting

The Mindfulness-Based Childbirth and Parenting (MBCP) protocol was created in 1998 by Nancy Bardacke, a nurse, midwife, and mindfulness trainer. Through participation in the MBCP protocol, extrapolated from Kabat-Zinn’s [15] Mindfulness-Based Stress Reduction (MBSR) protocol, pregnant women and their partners have the opportunity to learn mindfulness techniques to cope with the states of anxiety and stress ordinarily present during pregnancy, pain, and fear during childbirth, as well as to develop couple cooperation and parenting sensitivity [24,25,31]. MBCP was initially a group course held for 3 hours once a week for 9 weeks. The 9-week MBCP course includes a day of silent meditation between weeks 6 and 7 and an additional reunion gathering after all women have given birth. Participants begin MBCP in the late second or early third trimester and are asked to commit to home practice using guided meditation CDs for 30 minutes a day, 6 days a week throughout the course. Formal mindfulness meditation instruction is given in each class, including sitting meditation, body scan, mindful yoga, and loving-kindness meditation, all tailored to include attention to mental and physical aspects of pregnancy (eg, noting fetal movements during the body scan) [24]. For a description of the main topics covered week by week, refer to Multimedia Appendix 1.

The literature indicates the extraordinary effectiveness of the MBCP protocol both during [23,32-36] and after pregnancy [20,37-39]. Creswell [22] found a moderate effect size (Cohen *d*=0.5) for mindfulness interventions in reducing anxiety and depression in pregnant women. Duncan et al [32] reported a pooled effect size of *d*=0.48 for mindfulness-based interventions in enhancing psychological well-being during pregnancy.

Some of the benefits of mindful birthing are (1) an increase in confidence and decrease in fear of childbirth; (2) tapping into deep inner resources for working with pain; (3) improving couple communication, connection, and cooperation; and (4) providing stress-reducing skills for greater joy and well-being [40].

Results suggest the effects of a group-based psychosocial intervention during pregnancy may endure for years, well beyond the initial perinatal period. Investing in prevention and intervention efforts for mental health during pregnancy may have long-term benefits for the well-being of women [39].

There are also several potential benefits to participating in a mindfulness program individually, especially during pregnancy. Participants can choose when and where to practice, making it easier to incorporate mindfulness into a busy schedule or daily routine. Practicing mindfulness alone can also help women concentrate more deeply on their thoughts, feelings, and sensations without distractions from group dynamics, encouraging deeper self-reflection and self-awareness, fostering a better understanding of personal experiences and emotions. For those who may feel anxious in group settings, individual participation can provide a more comfortable environment to explore mindfulness. Individuals can also select specific practices or techniques that resonate with them, creating a more personalized experience; this can empower participants to develop their mindfulness skills independently, fostering a sense of ownership over their mental well-being.

There are several digital apps that are based on MBCP principles. For example, Mindful Mama (Mindful Mamas Club) [41], an app that provides resources and guided meditations designed to support mothers during pregnancy and parenting, incorporating mindfulness techniques, or Mind the Bump (Smiling Mind) [42], which offers mindfulness practices, educational resources, and a community support feature for parents-to-be, integrating MBCP concepts. None of these involve the use of an online coach.

### Our Study

MBCP intervention, which has already been validated and tested, will be fully available to users through digital tools. In particular, it will be delivered by a mobile app and guided by an online assistant, MAIA (developed by FBK). This research aims to assess the prototype of the MAIA app. This paper describes the prototype technology-based mindfulness intervention’s design and development process for pregnant women, including the exploration phase, intervention content development, and iterative software development (including design, development, and formative evaluation of paper- and low-fidelity prototypes).

## Methods

### Overview

The design and development process encompassed a multidisciplinary approach and continuous, systematic evaluation throughout, as recommended in the Center for eHealth Research and Disease Management comprehensive roadmap approach to improve the uptake and impact of eHealth technologies [43].

The study’s principal investigator (SR), a researcher and psychologist, led the intervention development work. During the design and development phase, the multidisciplinary project team, consisting of psychologists, mindfulness experts, communication experts, and information technology developers, had biweekly meetings. User-centered and service design methodologies were applied to ensure user involvement throughout the design and development process.

**Figure 1:**
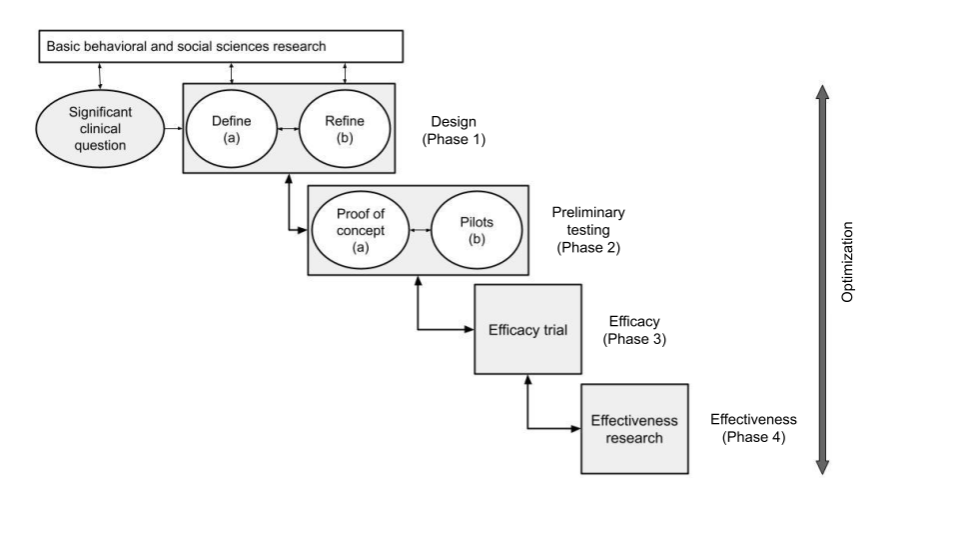
The ORBIT model.

**Figure 2:**
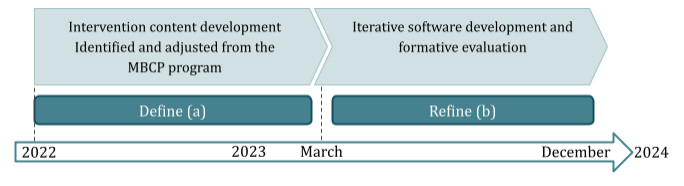
Development and iterative processes of the mindfulness intervention (Phase 1 of the ORBIT model—Design). MBCP: Mindfulness-Based Childbirth and Parenting.

The mindfulness intervention was developed in iterative processes, following the ORBIT model [44], as shown in Figure 1, through a combination of (1) intervention content development (identified and adjusted from the MBCP program) and (2) iterative software development of a prototypes and formative evaluation. The development and iterative processes of the mindfulness intervention are shown in Figure 2.

### Intervention Content Development

Adapting the protocol into a digital format involved an initial phase of studying the literature and the protocol itself. Once the main themes being presented week by week were identified, a team of psychologists with communication skills worked to adapt the content into an individual digital format delivered by chatbot. First, the team developed the online coach’s script and then multimedia content (videos, audio, and images) useful for delivering critical information in a way that is easier for women to understand than just text format.

The initial intervention content for this study was first developed by the primary investigator, then adapted and tailored by the entire research team through iterative processes to fit an 8-session-based intervention in electronic format through text, audio, video, and images. The intervention was user-tested to make the content easily accessible, confirm adaptation to an app format, and ensure that the scientific foundation for the intervention was intact. Table 1 lists and briefly describes the 8 sessions and the topics covered in each session.

The intervention was constructed in such a way that MAIA is always the first to interact with the user. It is currently not possible for the woman to ask specific questions. The chatbot provides information but also asks questions to help the user in self-reflection. The user’s response text is not analyzed at this stage of the study as it is not useful for the purpose of the study.

**Table 1:** MAIA intervention overview of sessions (8 sessions preceded by an introductory one) and their content divided into formal practices, informal practices, and readings or homework to be carried out independently between sessions.

Session	Formal practices	Informal practices	Readings or homework
S0	Welcome	Mindfulness definition. Formal practice and informal practice. Rituality, posture, and intentionality.	Preparation of materials and space
S1	Awareness of breath Raisin meditation	Being with the child	—^a^
S2	Body scan Awareness of breath	Mindful eating	“The Body scan”
S3	Body scan Awareness of breath	Mindfulness in daily life (brushing your teeth, dishes, cooking, and so on)	“Mindfulness in daily life,” “The pain of childbirth,” enjoyable events calendar
S4	Body scan Awareness of breath Mindfulness meditation-related pain	Informal pain practice	Calendar of unpleasant events, “Mindfulness practices for being with pain”
S5	Body scan Awareness of breath Mindfulness meditation-related pain Mindful touch-care meditation	Physical activity and informal practice, being with the child	—
S6	Body scan Sitting meditation Walking meditation	—	BRANN^b^ (make informed decisions) and Mindfulness day planning
S7^c^	Body scan Sitting meditation Loving-kindness meditation	Inquiry families of origin	—
S8	Personal choice (choose one or more meditation) Loving-kindness meditation	Continue informal practices	“Breastfeeding” and Mindfulness slogans, tips for continuing on your own

^a^Not applicable.

^b^BRANN: Benefits, Risk, Alternatives, Nothing, Now.

^c^A day of mindfulness practice was held between S6 and S7.

### Iterative Software Development and Formative Evaluation

Adapting the MBCP interventions by implementing the MAIA chatbot provides a novel approach to delivering psychological support. Users can engage in a comprehensive and effective intervention through personalized sessions. Specifically, in the initial session, users are asked to select 2 days of the week on which they want to be contacted by MAIA to conduct mindfulness sessions, along with their preferred time slot (morning, afternoon, or evening). In addition, on the basis of some answers given, MAIA provided them with customized information (eg, MAIA asks whether or not the user wants to do a review of the previous session, whether they prefer to do a certain mindfulness practice now or later, and so on and on the basis of the answers the dialogue tree takes different directions). Therefore, considering different sections of the app that could be integrated was critical to supporting user engagement, adherence, and overall well-being.

In the first iteration, in spring 2023, 2 psychologists and 2 communication experts tested preliminary. They gave feedback on the prototype to ensure that the intervention program was logically built.

**Figure 3:**
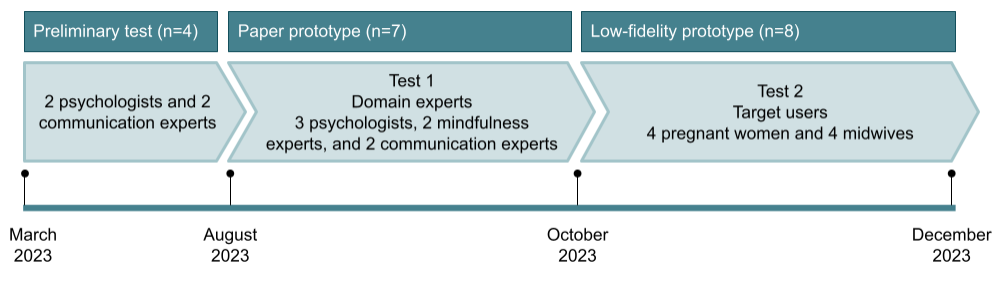
Timeline of software development and formative evaluation specifying the people involved (N=19).

At this co-design stage, the involvement of experienced professionals, in addition to end users, is of paramount importance. For this reason, 3 other psychologists, 2 mindfulness experts, and 2 communication experts were involved in a later study (test 1) by creating a paper prototype. Later, after the development of a low-fidelity prototype, end users (4 women and 4 clinicians) were also involved (test 2). Thus, a multistage evaluation of the MAIA prototype was conducted between August and December 2023, involving 15 participants, as elaborated in Figure 3.

### Identification of Variables

To gather the necessary information from tests 1 and 2 with the paper prototype and the low-fidelity prototype, key variables were identified for investigation—communication, session structure, materials, and content. Given the study’s objectives, a list of subvariables was chosen to be created ad hoc for the research. Table 2 shows the chosen variables and their respective subvariables. It was also decided not to ask each person everything. Still, based on the domain, a grid was created to determine which subvariables to investigate with the people involved.

**Table 2:** List of variables and subvariables investigated and people involved in evaluating individual variables.

Variables and subvariables		Psychologists	Mindfulness experts	Communication experts	Target	Midwives
**Communication**						
	Empathy and listening	✓	✓	✓	✓	✓
	Smoothness and fluidity	✓	✓	✓	✓	✓
	Chatbot interaction	✓	—^a^	✓	✓	✓
	Lexicon	✓	✓	✓	✓	✓
**Session structure**						
	Interaction length	✓	✓	✓	✓	✓
	Notifications and reminders	✓	—	✓	✓	—
	external links	—	—	✓	✓	✓
**Materials**						
	Diary	✓	✓	✓	✓	✓
	Audio tracks	✓	✓	✓	✓	✓
	Infographics and videos	✓	✓	✓	✓	✓
**Content**						
	Adherence to MBCP^b^ protocol	—	✓	—	—	—
	content evaluation	✓	✓	—	✓	✓
	content clarity	—	✓	✓	✓	✓

^a^Not applicable.

^b^MBCP: Mindfulness-Based Childbirth and Parenting.

These variables were assessed through the semantic differential tool [45] and a semistructured interview. The semantic differential is an instrument consisting of a series of scales, each composed of a pair of bipolar adjectives between which a rating scale (generally 5- or 7-position) is placed.

Interviews, instead, are suitable for a more in-depth investigation of users’ attitudes

and preferences toward new technological solutions since open-ended discussions with users can help researchers better understand the issues and concerns related to the possible future adoption of these solutions.

### Iterative Development and Prototypes

Based on needed content adjustments, the first prototype version of the software was developed. This initial paper prototype consisted of the scripted dialogue of the chatbot and screens presenting the different sections of the app. All professionals involved were assigned specific days of the mindfulness sessions to view. They were then asked to complete an online questionnaire based on the semantic differential. In this study, we used a 4-point rating instead of 5 or 7 points to prevent neutral feedback allocation. The rating scale displayed semantically opposing adjectives with negative connotations on the left and positive connotations on the right, representing the extremes. The pairs of adjectives chosen explicitly for this study correspond to 1 of the 4 macrovariables under investigation. Participants were tasked with indicating, for each adjective pair, which adjective better characterized the specified variable related to the chatbot. For instance, if the adjective pair is “passive listening-active listening,” associated with the “communication” variable, participants would choose point 1 or 2 if they perceive the chatbot’s listening as passive and point 3 or 4 if they perceive it as more active. Following the questionnaire, a short semistructured interview was conducted to investigate participants’ impressions. The interviewer posed specific questions, and then an opportunity was given for the interviewee to share additional open reflections. To facilitate subsequent analyses, the interview was recorded after the participant’s direct consent was obtained. Multimedia Appendix 2 shows the list of topics and questions posed during the interviews.

To recruit participants, we adopted criterion-based sampling. Specifically, participants had to be psychologists, mindfulness instructors, or communication experts. Participants were recruited through instant messaging tools (WhatsApp [Meta]) and email.

In the first iteration, psychologists, mindfulness, and communication experts tested and gave feedback on the prototype to ensure that the intervention program was logically built. Participants were given oral and written information about the study and provided written informed consent before user testing.

At this stage, the Personas methodology was also found useful. Personas is a user-centered and service design method used to create and visualize fictional representations of the target group [46]. Personas is an effective method for all project team members to understand the target group for which the app is built better. The personas in this study contained information about the pregnancy background, challenges, and technology use. Refer to Multimedia Appendix 3 for illustrated examples of study Personas. Participants had to identify with one of these Personas before the reading.

The data obtained from the administered questionnaires underwent aggregated analysis. Therefore, participants’ evaluations on a 4-point scale for each adjective pair were summed. Once summed, the arithmetic mean for each adjective pair’s evaluation was calculated. After obtaining all the average scores, a dot chart was generated using Datawrapper (Datawrapper GmbH), a web-based charting and mapping tool [47], to observe the overall trend of the scores better and, consequently, the semantic pole toward which they tend to orient.

The information obtained during the interviews has been transcribed for a more detailed analysis. The respondents’ answers were then categorized by topics within the 4 initially identified variables—communication, session structure, materials, and content. In addition to these variables, a new theme emerged concerning the “postintervention” phase. The categorized responses within the variables were further divided into their respective subvariables (for the complete list, refer to Table 2). Finally, they were classified into positive and negative aspects or suggestions (areas for improvement). By “suggestions,” we refer to those aspects of the app that might need modification in a forward-looking perspective.

Following minor adjustments, the paper prototype was transformed into an electronic tool to simulate the chatbot and app concept. The intervention was delivered through a software platform called TreC-PerseO (FBK), which supports the delivery of digital behavior change interventions. This proprietary software architecture is designed to ensure privacy and certified workflows in a production environment while also facilitating prototyping by adapting to the steps outlined in the chosen methodology. In this phase, Telegram (Telegram Messenger Inc) chat was used to deliver the intervention (to validate the content without the need to collect personal user data). During this second iteration (test 2), the final version of the low-fidelity prototype was tested by 2 pregnant women, 2 mothers, and 4 midwives. The participants were assigned different days of the mindfulness sessions to review, after which they completed an online questionnaire based on the semantic differential. A short interview followed the questionnaire.

To recruit participants, we adopted criteria-based sampling. Specifically, participants had to meet the following criteria: be pregnant or mothers with children aged less than 1 year or be midwives. Participants were recruited through instant messaging tools (WhatsApp) and email. The sample collected was 8 participants—2 pregnant women, 2 mothers, and 4 midwives. Participants were given oral and written information about the study and provided written informed consent before user testing.

The data collected during the interviews has been transcribed for a more detailed analysis. The respondents’ answers were then categorized by topics within the 4 initially identified variables. The categorized responses within the variables were further divided into their respective subvariables, and then they were classified into positive and negative aspects or suggestions (areas for improvement). The resulting participant’s feedback data were again used to evaluate, refine, iteratively adjust, and upgrade the prototype.

### Ethical Considerations

At the time of enrollment, all participants who freely decided to take part in the study were asked to sign an informed consent form after a careful explanation of the study, its aims, the level of involvement required, and the duration of the research, as well as all ethical issues concerning confidentiality. All data collected were anonymized before analysis.

The participants will then be informed of the study’s results through the dissemination of articles.

In line with Italian Law No. 3/2018, ethical review is mandatory for clinical trials, therapeutic procedures, or studies involving health risks or sensitive data. This study, being nonclinical and focused on prototype development, did not meet these criteria, justifying the absence of ethics committee involvement.

No compensation was provided for participants.

## Results

### Intervention Content Development

The iterative content development processes were parallel to app programming, and adjustments were made based on usability testing. Therefore, the app is designed to include 5 sections: the chatbot, Practices, Gallery, To Do List, and Diary.

**Figure 4:**
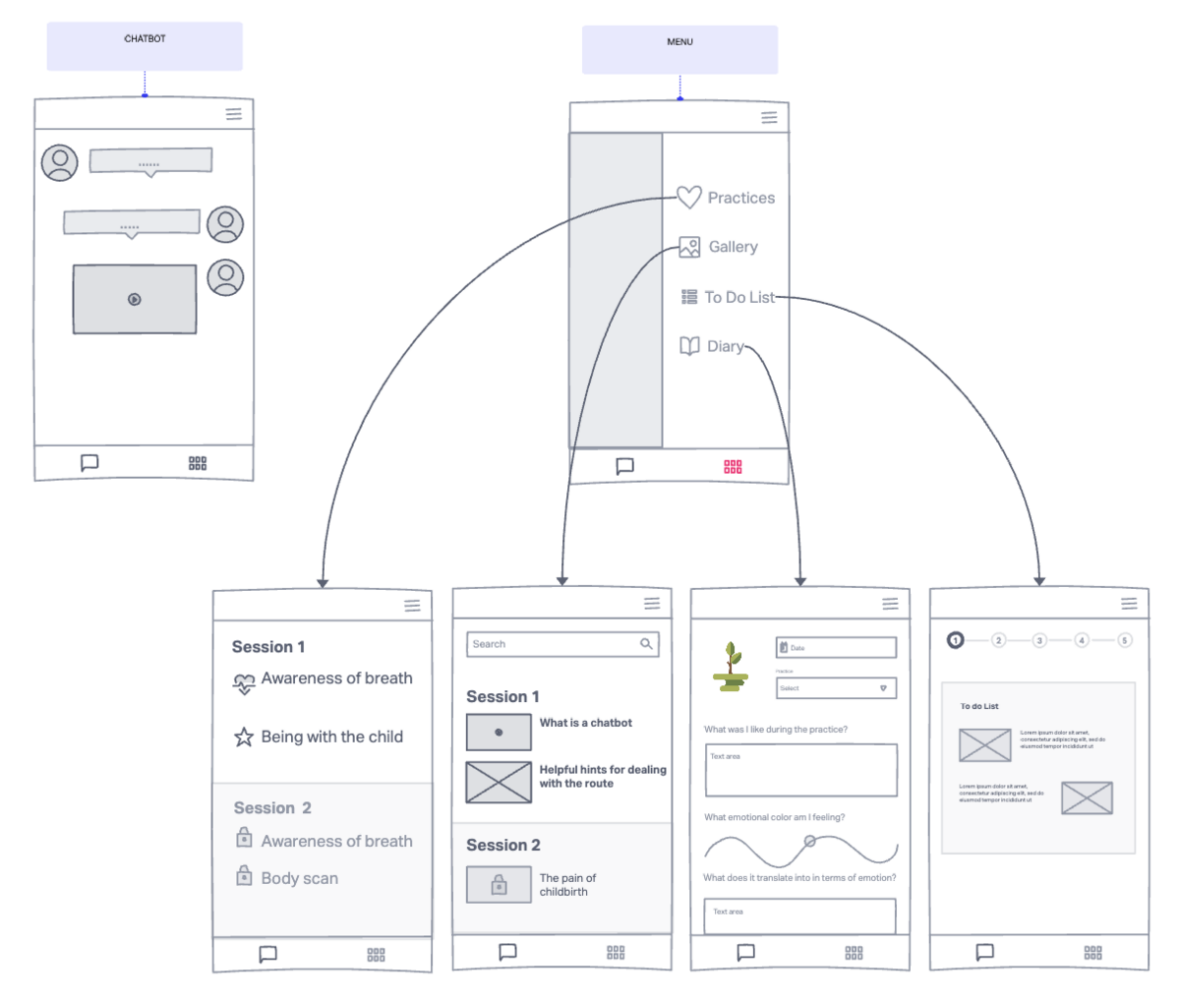
Mockups of the MAIA app with its different sections.

Figure 4 shows some mockups of the app with its different sections.

MAIA assigns homework during the session that the users should carry out during the subsequent days, so the app Diary section will support self-monitoring. We have also provided for a tree that grows as the woman progresses. All the exercises women need to do during the week are listed in the To-Do List section to help her remember what she needs to do. All the practices, divided week per week, are shown in the Practices section. The Gallery page features all multimedia material delivered by MAIA, such as educational videos, images, and intervention introductions.

### Iterative Software Development and Formative Evaluation

#### Iterative Development and Preliminary Test

A total of 2 psychologists and 2 communication experts took part in this preliminary test phase. The objective of this evaluative phase was to test, through role-playing, the communicative style of dialogues—empathetic, welcoming, fluent, and smooth.

Therefore, it was possible to evaluate and revise MAIA’s communication style through role-playing.

**Figure 5:**
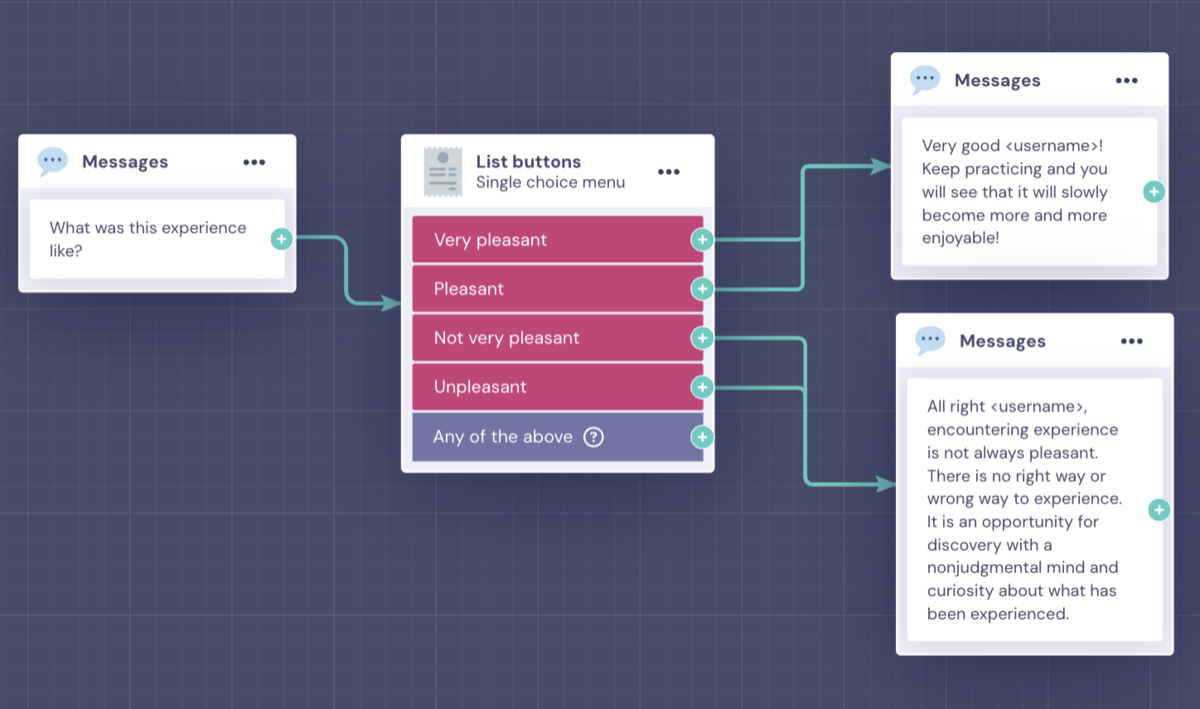
Examples of new multiple-choice questions included after preliminary tests.

Regarding fluency and smoothness, the evaluation was primarily positive. To make the protocol more and more realistic, the dialogues were revised in some parts. Changes were made grammatically and syntactically to make the text more fluent and read smoothly. The changes also considered empathy. This makes it possible to create a feeling of trust in the relationship. Through empathic communication, the person can feel in a safe space, within which he or she is reassured and protected. Initially, the feeling while reading the dialogues could have been more apathetic communication. Therefore, multiple-choice questions were included about the individual practice (eg, How was this experience?) and the general course of action (eg, How did you do with mindfulness practices last time?). This allows MAIA to respond appropriately and more accurately based on the user’s experience. Some examples are illustrated in Figure 5.

A new practice not provided for in the MBCP initiate protocol, namely the practice of 3 breaths before each session, was also included. The user receiving notification from MAIA can be anywhere and under any conditions. To help the person focus on the present moment and be ready for the practices offered later, it was decided to provide this new initial practice at each session.

Finally, the correctness of the videos and images being sent was checked. For each video link, consistency was checked against the instructions, and the topic was addressed. Everything was correct, so no changes had to be made.

#### Iterative Development and Paper Prototype

After modifying some parts of the dialogue, as described above, the first paper prototype was developed. In this phase, the sample involved consisted of 7 professionals—3 psychologists, 2 mindfulness instructors, and 2 communication experts.

Using a semantic differential-based questionnaire facilitates observing the respondents’ average positioning with respect to the 4 macrovariables under investigation.

**Figure 6:**
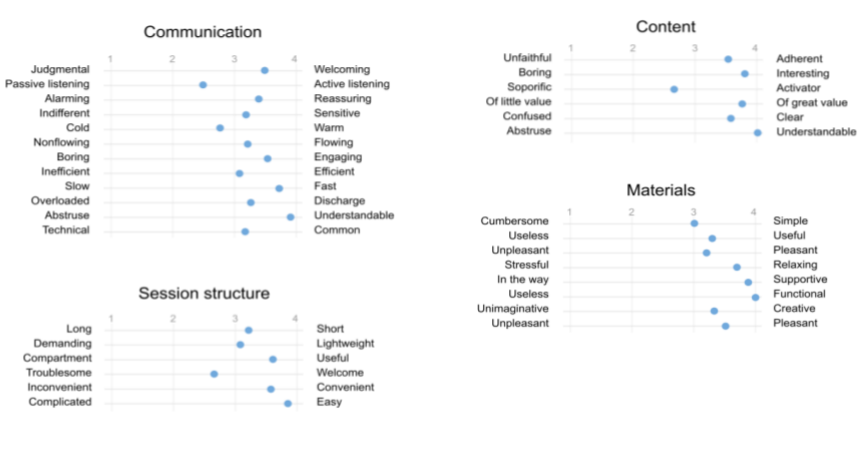
Graphical representation of mean values derived from semantic differential administered to domain experts (psychologists, mindfulness instructors, and communication experts).

As can be seen from Figure 6, a clear tendency toward the positive semantic pole (right pole) emerges in the feedback. This means that a significant portion of the chatbot-related feature pairs were evaluated in the positive rather than the negative sense. For instance, the vocabulary used by the chatbot was judged as understandable rather than abstruse. However, feedback related to 4 pairs of features occupied a middle position between the positive and negative poles, that is, passive listening–active listening, cold-warm, troublesome-welcome, and soporific-activator.

In total, 2 psychologists (experts in communication) conducted the qualitative interview to gather additional information. Table 3 briefly highlights key positive and negative aspects that emerged during the domain experts’ interviews.

In particular, the analysis of the interviews unearthed several notable observations that garnered attention due to their recurrent mention by more experts. Certain interviewees advocated augmenting written textual content with an auditory counterpart or substituting it entirely with auditory or audiovisual formats. The diary, initially conceived as a personal and confidential tool, prevents external control. However, the process of diary entry may elicit personal experiences, necessitating potential consultation with a designated professional if the need arises.

A further debated aspect is the inclusion of background music in mindfulness exercise tracks. According to certain interviewees, such music might prove distracting, diverting attention away from the individual’s introspective focus. Conversely, others contend that it could serve as an aid, particularly in the absence of direct human interaction. In summation, these insights are deemed fundamental for the following stages of research, wherein the perspectives of pregnant women directly engaged in this study can be further investigated. In addition, we underscore the presence of numerous positive aspects delineated in the interviews, including the empathetic and welcoming communication style, the fluidity of discourse, and the use of imparted content, among others.

**Table 3:** Positive and negative aspects emerged from interviews with domain experts (psychologists, mindfulness instructors, and communication experts).

Variables and subvariables		Positive aspects	Negative aspects (and suggestions)
**Communication**			
	Empathy and listening	Empathic communication is comprehensive, conveys calm, reassuring, warm, welcoming, and puts at ease Nice dialogue, relaxing, helpful Customized	Include more sentences that also address the child Responses are repetitive and somewhat mechanical “How did it go (the process)?” is too judgmental
	Smoothness and fluidity	Very smooth Enjoyable to read	—^a^
	Chatbot interaction	If I read, I can go at the speed I want It is not artificial, it works	Needs to be more engaging Reading can be tedious Audio-only or audio and video or written and audio
	Lexicon	Accessible to everyone: intuitive, simple, straightforward, and understandable Gentle, suitable, and consistent terms	The term “discomfort” may cause concern The term “pain” is repeated
**Session structure**			
	Interaction length	Not demanding Self-management of dialogue times	—
	Notifications and reminders	Helpful in saving and reviewing it without entering the app	—
**Materials**			
	Diary	Putting into writing what is felt allows for processing and gaining clarity	Option to print the diary and write it by hand
	Audio tracks	Useful content “Being with the pain”: very helpful for preparing for childbirth Use of different voices	Banal video graphics Remove the music Add music Lack of initial welcoming moment Option to choose a male or female voice
	Infographics and videos	Beautiful graphics, practical, and charming Nice color used Option to print PDFs	—
**Content**			
	Adherence to MBCP^b^ protocol	Positive and negative experiences of other women Liked the 8 attitudes	Providing two options (same content presented differently) based on past pregnancy experiences
	Content evaluation	Interesting content Practices dedicated to both mother and baby	Having too much time to think is only sometimes positive
	Content clarity	Engaging protocol	—
Other		—	Understand how it went postpartum in light of these weeks Hold an in-person meeting at the end of the protocol to make it more engaging

^a^Not available.

^b^MBCP: Mindfulness-Based Childbirth and Parenting.

#### Iterative Development and Low-Fidelity Prototypes

**Figure 7:**
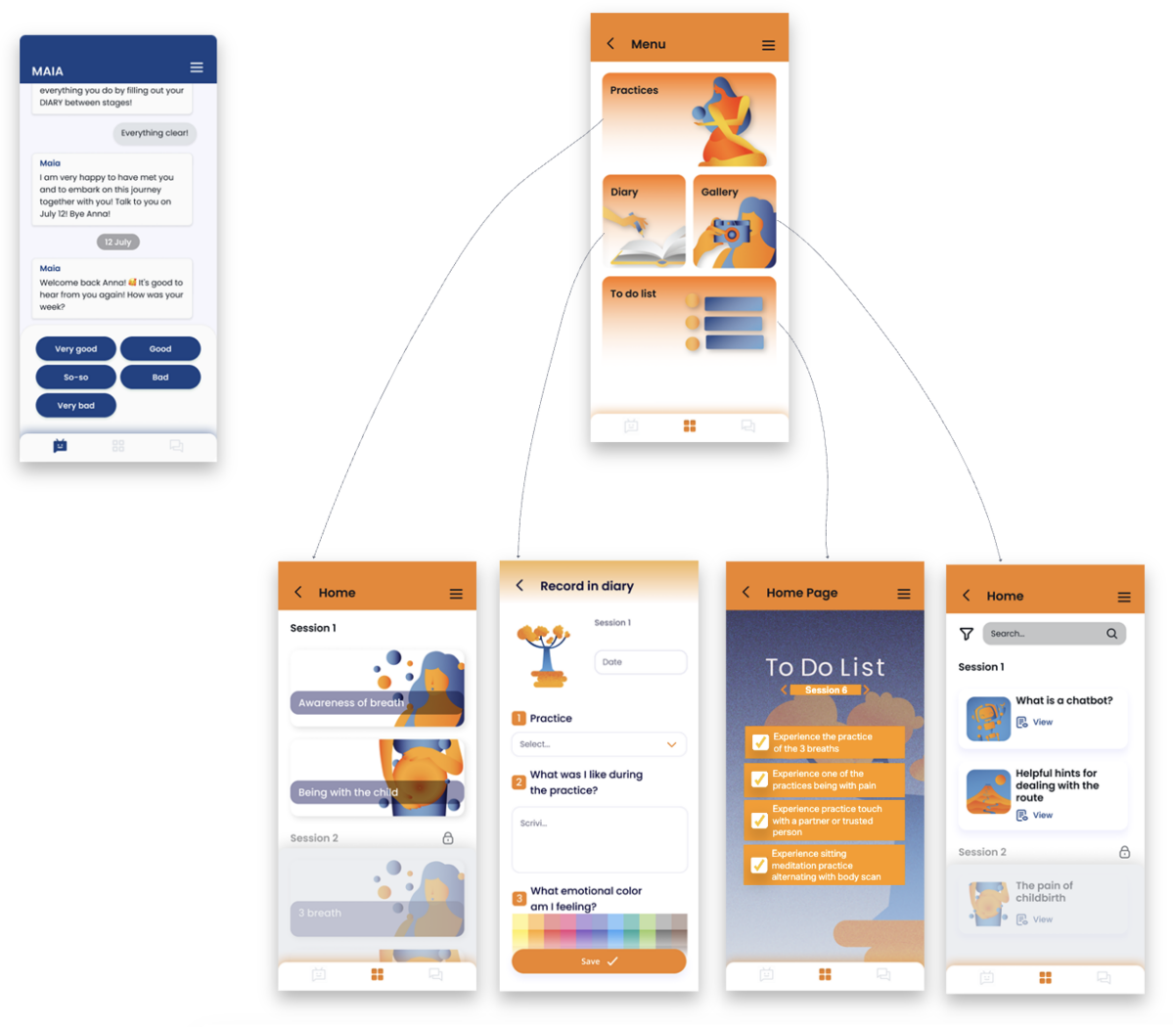
More realistic mockups of the MAIA app with its different sections.

In total, 2 pregnant women, 2 moms, and 4 midwives participated in this second test phase. To better understand the app and the entire intervention, more realistic mockups were created (Figure 7). This allowed users to represent the app in its final appearance better.

In this case, using a semantic differential-based questionnaire facilitates the observation of the respondents’ average positioning with respect to the 4 variables under investigation.

**Figure 8:**
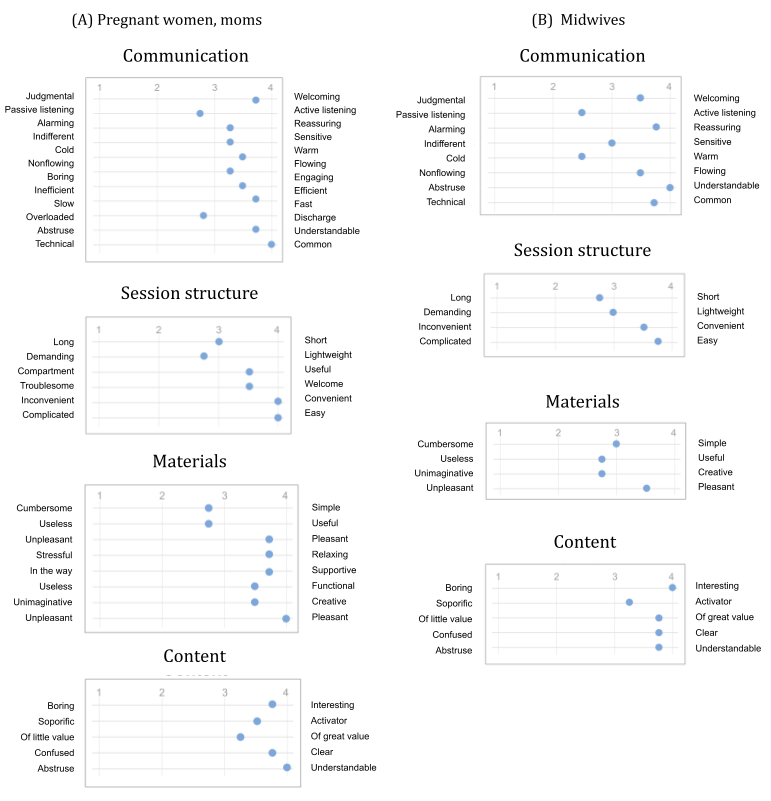
Graphical representation of the mean values obtained from administering the semantic differential to target users.

As illustrated in Figure 8, a tendency toward the positive semantic pole (right pole) emerges in the feedback also in these groups of participants. However, feedback related to 5 pairs of features occupied a middle position between the positive and negative poles for the pregnant woman and moms’ group and 5 for the midwives group.

Furthermore, 2 psychologists (experts in communication) conducted the qualitative interview to gather additional information. Table 4 briefly highlights key positive and negative aspects that emerged during user interviews.

All respondents found the protocol content of great value, considering it interesting and useful. They appreciated the alternation of simple and streamlined proposals with more time-consuming ones. They consider it an added value to manage the timing of the course autonomously; with the app, they can postpone the performance of activities, reassured by the fact that they can catch up at another time. They also mostly consider the diary to be useful and usable as long as it is left as an option.

One of them suggested supplementing the stand-alone activities with a couple of face-to-face meetings (an initial one to explain the meaning and experience as a group and a central one for the more engaging exercises, such as the sultana and ice cube exercises).

The midwives dwelt more on the contents and found them correct and set out in a simple and comprehensible manner for all, giving useful advice to improve the video part in a couple of cases. One of them strongly urged the translation of the protocol into several languages.

Among the materials, the infographics were highly appreciated by both categories for their incisiveness and the fact that they were printable.

**Table 4:** Positive and negative aspects emerged from user interviews (pregnant women, moms, and midwives).

Variables and subvariables			Positive aspects		Negative aspects (and suggestions)
**Communication**					
	Empathy and listening	Nice dialogue Communication puts at ease Customized, nonjudgmental		Responses are repetitive, somewhat mechanical When the exercise does not work, it could be a question that goes into depth analyses and also advises for future exercises	
	Smoothness and fluidity	Very smooth Enjoyable to read		—^a^	
	Chatbot interaction	It is not artificial, it works It is fast and light, particularly when it proposes questions with multiple-choice answers		—	
	Lexicon	Accessible to everyone: intuitive, simple, clear, and understandable		—	
**Session structure**					
	Interaction length	Not demanding Self-management of dialogue times		—	
	Notifications and reminders	Useful even if you have no time at that moment		Sometimes stressful because even at the scheduled time, you cannot do the exercises	
**Materials**					
	Diary	Useful if optional Interesting to focus on different exercises Very useful to promote awareness		It depends on the available time	
	Audio tracks	Useful content Good timing Good voice		Add music Sometimes they are too long	
	Infographics and videos	Beautiful graphics, effective, and charming Very useful Infographics to focus on the most important information, and to repeat the exercise Very useful images in the videos, especially for people with visual memory		The pregnant woman is too thin The eighth-week video on pain has a very heavy atmosphere and does not reflect the real situation in the delivery room (the bed is used very little, and the woman is never alone)	
**Content**					
	Content evaluation	Interesting content Engaging protocol Very valuable content The content on breastfeeding was very well done, it says the right things in the right way The daily practical exercises are very interesting and engaging (eg, walking and teeth washing) It is engaging because the commitment required is growing The practice of three breaths is very useful and effective		—	
	Content clarity	Very clear and simple to follow		At first, it seems like the practice will always last 5 minutes	
Other			Very comfortable to have all you need in the app		Desire to have other two weeks after the childbirth Hold an in-person meeting at the beginning and at the end of the protocol to make it more engaging It could be easier to follow the most demanding exercises in the team, with a guide

^a^Not available.

## Discussion

### Principal Findings

This study illustrates how user-centered and service designs can be applied to identify and incorporate essential stakeholder aspects in the design and development process. Combined with evidence-based concepts, this process facilitated the development of a mindfulness intervention designed for the end users, in this case, pregnant women.

Importing and adapting the intervention was a comprehensive and meticulous process. According to the ORBIT model, we examined a wide range of scientific studies on the efficacy of mindfulness during pregnancy and the postpartum period, focusing on the MBSR and the MBCP protocols. By integrating elements from both methodologies, we tailored key aspects of each protocol to create an intervention specifically designed for the needs of pregnant women. In addition, we considered other evidence and best practices in the field of mindfulness, ensuring the incorporation of empirically supported and culturally sensitive approaches (ie, the practice of the 3 breaths to anchor oneself in the present moment).

This integrative approach allowed us to develop a protocol that is scientifically grounded, practical, and suitable for the unique context of pregnancy.

Paying attention to the semantic differentials with lower values, we can see that in the case of listening, the answers of the users and midwives agree. In the interviews, 3 out of 4 women stated that they see the chatbot as the necessary tool to dispense material. Furthermore, 2 of them consider the dialogues to be valuable when it is fast and nondemanding (eg, when a multiple-choice answer is expected), which is also confirmed by the results on the overloaded or discharged differential. However, as seen from the other semantic differentials, the chatbot is welcoming, reassuring, and polite.

Consider the pair “passive listening–active listening,” referring to whether one feels actively or passively listened to by the chatbot; domain experts, on average, did not distinctly categorize the listening as active. This judgment may be due to the inherent limitation of digital apps—lacking a natural person on the other side of the screen responding to each user’s comment. The structure of this type of interaction, therefore, makes it difficult to feel fully heard, particularly in an active way.

One of the suggestions we received from users was to add audio in addition to the written text. This suggestion stems from sociocultural considerations. Indeed, nowadays, people increasingly use social networks, interacting with content that is short in duration and predominantly in the form of video rather than text. Reading may, therefore, appear more boring for some. Another focal point pertains to the diary feature, particularly the need to facilitate interaction with a designated professional.

### Strengths, Limitations, and Future Directions

One of the main strengths of this study lies in the fact that it engaged both domain experts and target users from the very early stages. The suggestions that were gathered highlight different but equally important points of view for refining the intervention. Having midwives involved, in addition to pregnant women, once again allowed us to have an additional point of view. The midwives could give us suggestions (eg, editing a video so that it was more aligned with what is happening in the delivery room) that none of the other actors involved would have been able to provide.

One limitation, however, mainly concerns the development of the low-fidelity prototype and the number of users involved. Along with that prototype, we provided users with a mockup of the app, but interacting with such a prototype or with the final app is a very different experience. The purpose of this study, however, was not to evaluate the user experience with the app but to gather impressions of content, vocabulary, and communication style. For that reason, the tool used was deemed good enough. The next step will be to develop the application following the suggestions gleaned from this first study and then to set up a proof-of-concept study, following the orbit model, involving about 50 women to evaluate the acceptability, feasibility, and usability of the MAIA app [48].

Based on the data collected during this second study, we expect to have to make further modifications to the intervention and application. Following these modifications, we will finally be ready to field a randomized control trial to evaluate the efficacy of the intervention (the study protocol will be finalized by mid-2025). After conducting this randomized controlled trial, we also plan to conduct a real-world study in order to evaluate the effectiveness of the intervention and the app itself.

### Conclusions

Developing a digital intervention is a complex procedure that requires work in different areas and involves different professionals. We aimed to build an intervention that was as suitable as possible for the target audience, both in terms of content and design. The contribution of specialists is undoubtedly an added value from the early stages in a coconstruction perspective. This survey is the first step in a series of studies to validate the intervention. At this stage, it is still difficult to state how this research contributes to the broader evidence base supporting the use of mindfulness-based practices among pregnant women, as we are still at a preliminary stage. However, we can state that the impression of the users and experts involved is positive; as the results seem to be encouraging, the next step will be to conduct a study to assess the experience and engagement of pregnant women.

## Appendix

Multimedia Appendix 1 Brief description of the main topics covered week by week in the Mindfulness-Based Childbirth and Parenting original program.

Multimedia Appendix 2 Questions posed to participants attending the evaluation.

Multimedia Appendix 3 Examples of personas.

## Abbreviations

MBCP: Mindfulness-Based Childbirth and Parenting
MBSR: Mindfulness-Based Stress Reduction

## Data Availability

The datasets generated and analyzed during this study are available from the corresponding author upon reasonable request. Before disclosure, data are aggregated and anonymized.
