# Supplementary figures and images for: A Mindfulness-Based App Intervention for Pregnant Women: Qualitative Evaluation of a Prototype Using Multiple Case Studies
Source: JMIR Form Res. 2025 Jan 17;9:e58265. doi: 10.2196/58265 (PMC11786139; doi:10.2196/58265)

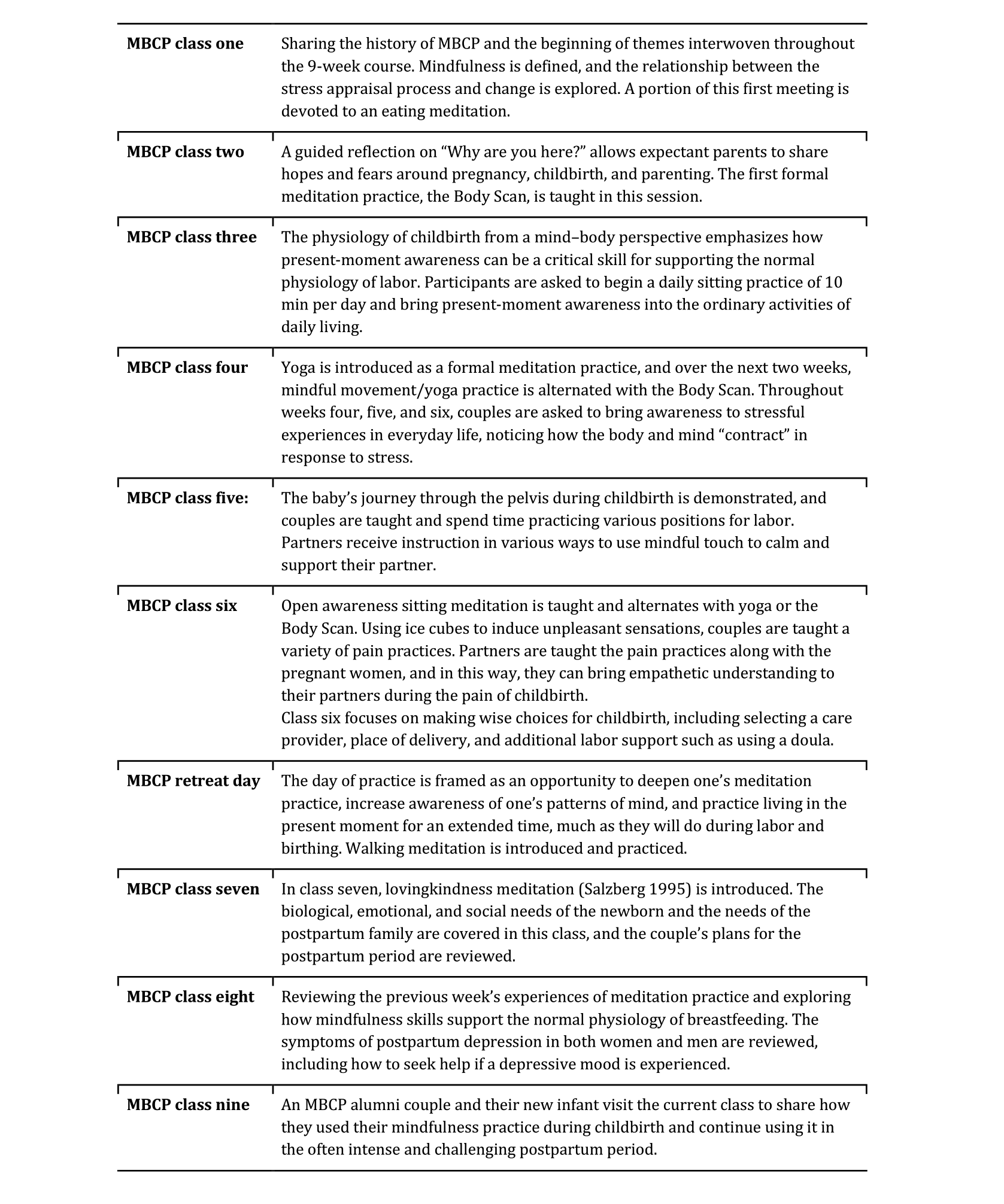

Supplement: Multimedia Appendix 1 [file formative_v9i1e58265_app1.png]

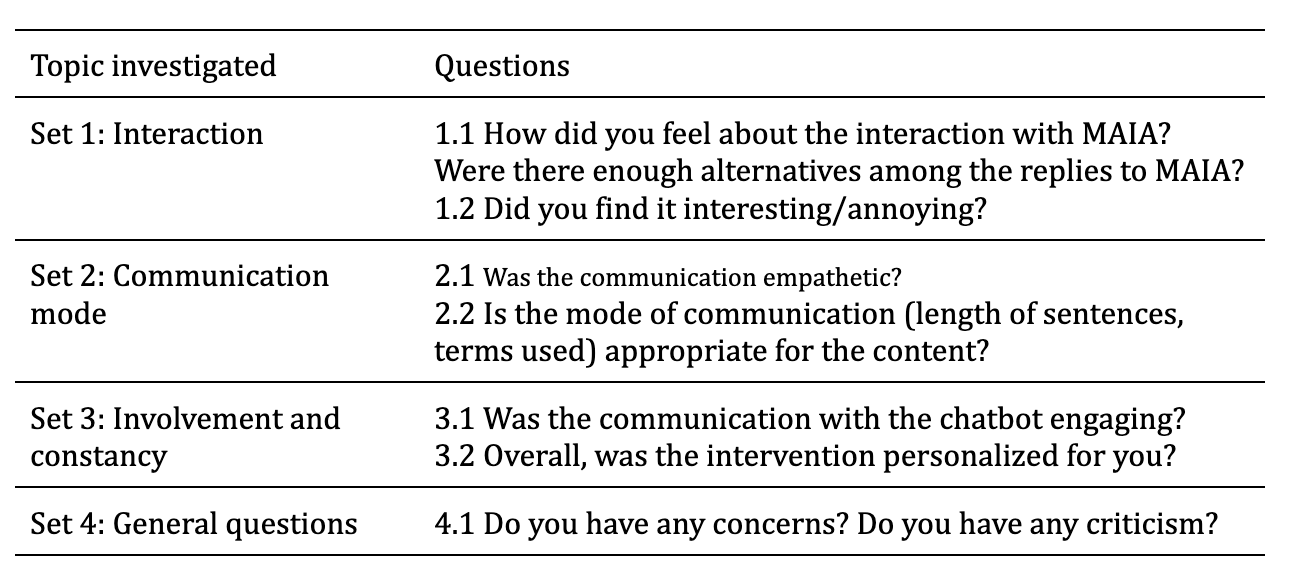

Supplement: Multimedia Appendix 2 [file formative_v9i1e58265_app2.png]

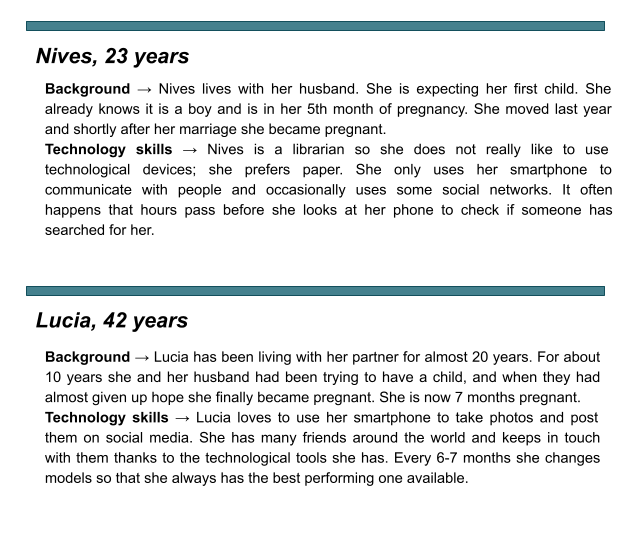

Supplement: Multimedia Appendix 3 [file formative_v9i1e58265_app3.png]
